# Establishment of *Aspergillus fumigatus*-specific IgG cut-off level for diagnosis of chronic pulmonary aspergillosis in the Iranian population

**DOI:** 10.22034/cmm.2026.345514.1741

**Published:** 2026-06-03

**Authors:** Sabrieh Asadi Shahi Sarae, Masoud Aliyali, Vida Mortezaee, Hamidreza Jamaati, Hossein Asgarian Omran, Siavash Abedi, Hossein Mehravaran, Jamshid Yazdani Charati, Maryam Sadat Mirenayat, Mihan Pourabdollah, Somayeh Lookzadeh, Maryam Moazeni, Sabah Mayahi, Mona Ghazanfari, Iman Haghani, Mahdi Abastabar, Mohammad T Hedayati

**Affiliations:** 1 Student Research Committee, School of Medicine, Mazandaran University of Medical Sciences, Sari, Iran; 2 Department of Medical Mycology, School of Medicine, Mazandaran University of Medical Sciences, Sari, Iran; 3 Pulmonary and Critical Care Division, Imam Khomeini Hospital, Mazandaran University of Medical Sciences, Sari, Iran; 4 Chronic Respiratory Diseases Research Center, National Research Institute of Tuberculosis and Lung Diseases, Shahid Beheshti University of Medical Sciences, Tehran, Iran; 5 Department of Immunology, School of Medicine, Mazandaran University of Medical Sciences, Sari, Iran; 6 Department of Biostatistics, Faculty of Health, Mazandaran University of Medical Sciences, Sari, Iran; 7 Pulmonary Rehabilitation Research Center, National Research Institute of Tuberculosis and Lung Diseases, Shahid Beheshti University of Medical Sciences, Tehran, Iran; 8 Invasive Fungi Research Center, Communicable Diseases Institute, Mazandaran University of Medical Sciences, Sari, Iran

**Keywords:** *Aspergillus fumigatus*, Chronic obstructive pulmonary disease, IgG

## Abstract

**Background and Purpose:**

The optimal cut-off level for serum *Aspergillus*-specific IgG (S*Af*IgG) in the diagnosis of chronic pulmonary aspergillosis (CPA) remains to be established for different countries. This study aimed to establish a cut-off level for S*Af*IgG in CPA diagnosis and validate its diagnostic accuracy in the Iranian population.

**Materials and Methods:**

Diagnostic performance of S*Af*IgG was evaluated in patients with chronic obstructive pulmonary disease (COPD) suspected of having CPA who had referred to the two reference centers for pulmonary diseases in Iran, during 2021-2023. Levels of S*Af*IgG were measured using an enzyme-linked immunosorbent assay. Optimal cut-off point for S*Af*IgG was determined using the receiver operating characteristics (ROC) curve and Youden's index and was subsequently validated.

**Results:**

In total, 30 (15.3%) out of 196 COPD patients were diagnosed with CPA. The ROC curve analysis yielded an area under the curve of 0.632 (95% CI: 0.421-0.843; *p*=0.114), with an optimal cut-off value of 23.5 U (sensitivity: 71.4%; specificity: 61.4%). The S*Af*IgG levels were significantly higher in CPA patients (median [IQR], 39 [31-55] U) compared with non-CPA patients (20 [15-24] U; *p*<0.001).

**Conclusion:**

The findings indicated that S*Af*IgG, with a cut-off value of 23.5 U, provided moderate sensitivity and specificity for screening and may be used in combination with the culture method to confirm the diagnosis of CPA in COPD patients. However, the variability in cut-off values across different populations highlights the need for standardized guidelines to improve diagnostic consistency and reliability.

## Introduction

Chronic pulmonary aspergillosis (CPA) is an important and emerging infectious disease, with an annual incidence estimated at 1,837,272 cases and approximately 340,000 (18.5%) deaths [1]. Occurrence of CPA is closely linked to underlying lung diseases, particularly chronic obstructive pulmonary disease (COPD), which involves chronic and progressive inflammation of the lungs [2-5]. In Iran, CPA is significantly under-recognized, and diagnostic capacity is limited.

Diagnosis of CPA is challenging due to its slow and insidious progression, nonspecific symptoms, variable radiological findings, and the limited sensitivity of mycological cultures. As a result, many cases go undiagnosed, particularly in resource-limited countries [2]. If left untreated, CPA can progress to a severe end-stage disease, significantly impairing quality of life and posing life-threatening risks [6]. *Aspergillus*-specific IgG has emerged as a key diagnostic tool for CPA due to its less invasive nature and improved sensitivity and specificity, compared to traditional methods [7, 8]. A recent guideline for CPA in low- and middle-income countries emphasized the importance of using the serum *Aspergillus*-specific IgG (S*Af*IgG) test [9]. However, there is limited evidence regarding the cut-off values for S*Af*IgG, and optimal cut-off levels may vary across different geographical regions [10].

Recently, the International Society for Human and Animal Mycology–allergic bronchopulmonary aspergillosis Working Group [10] recommended that S*Af*IgG be developed for specific populations, as has already been established for some countries, including India [11], Japan [12], and the UK [13]. Given these considerations and the lack of a defined cut-off level for S*Af*IgG in the Iranian population as a useful diagnostic parameter for CPA, this study aimed to establish a cut-off level for S*Af*IgG in CPA diagnosis and also validate its diagnostic accuracy in this population.

## Materials and Methods

This study was performed on patients with COPD who had referred to the two reference centers for pulmonary diseases in Iran, Masih Daneshvari Hospital (the Reference Center for Tuberculosis and Pulmonary Diseases of Iran, Tehran) and Imam Hospital (the referral center for pulmonary diseases in Sari, Mazandaran) during 2021-2023. Diagnosis of COPD was made following the Global Initiative for Chronic Obstructive Lung Disease guidelines [14].

Sputum samples were collected from all study participants. Additionally, 5 ml blood samples were obtained from the patients, and serum was stored at -80 °C for future use. The enzyme-linked immunosorbent assay (ELISA) method was applied to measure the S*Af*IgG in serum samples (Demeditec *Aspergillus fumigatus* IgG ELISA DEASPG0680, Germany). Detailed information on the sputum preparation, culture and identification of the *Aspergillus* species, and measurement of S*Af*IgG Level are accessible in our published paper [15].

Diagnosis of CPA was made according to the guidelines established by the European Society for Clinical Microbiology and Infectious Diseases/European Respiratory Society criteria [2].

### Ethics approval and consent to participate

Ethics Committee of Mazandaran University of Medical Sciences (code: IR.MAZUMS.REC.1400.313) approved the present research, and written informed consent was obtained from the patients.

### Statistical analysis

The optimal cut-off point for specific IgG against *A. fumigatus* was determined by the receiver operating characteristic (ROC) curve. Diagnostic performance of S*Af*IgG was evaluated by calculating sensitivity, specificity, positive predictive value (PPV), and negative predictive value (NPV), along with ROC curve analysis. The area under the curve (AUC) was reported with 95% confidence intervals. Non-normally distributed data were reported as medians and interquartile ranges (IQR). Moreover, diagnostic odds ratio (DOR), Euclidean distance, and product were calculated. Chi-squared test was used for the comparison of the diagnostic value of IgG for CPA. The Mann-Whitney U test and the t-test were employed to compare the groups statistically and assess any significant differences in the data. All statistical analysis was performed in the SPSS software (version 27.0.1). The significance level in this research was 0.05, and 95% confidence interval was calculated.

## Results

In total, 7 (3.6%) out of 196 patients with COPD 7 cases were positive for *Aspergillus* spp. by culture, of which 5 out of 30 (16.6%) cases were from the CPA and 2 out of 166 (1.2%) from the non-CPA patients. *A. fumigatus* was the most common isolate (3/7, 42.8%) followed by *A. flavus*, *A. tubingensis*, and *A. citrinoterreus* (1/7, 14.2%, each).

### S*Af*IgG cut-off level

Distribution of S*Af*IgG levels in patients with COPD is illustrated in Figure 1. S*Af*IgG levels ranged from 6 to 100 U (mean: 24.1 U). The ROC curve analysis (Figure 2) was utilized to establish the cut-off values of S*Af*IgG in the positive *Aspergillus* culture group. The area under the ROC curve was precisely determined as 0.632 (95% confidence intervals [CI]: 0.421–0.843, *p*=0.114). Following the application of Youden’s index, the optimal cut-off value of S*Af*IgG was determined at 23.5 U. The DOR was 4.062, summarizing the effectiveness of the test by combining sensitivity and specificity; higher values indicate stronger discriminatory power. The Euclidean distance (0.476) represents the distance from the ideal point (sensitivity: 100%, specificity: 100%) on the ROC curve, helping to select the cut-off that balances sensitivity and specificity. The product (0.442) indicates the maximum combined value of sensitivity and specificity. These metrics collectively provide a quantitative and clinically interpretable assessment of S*Af*IgG performance for CPA diagnosis.

**Figure 1:**
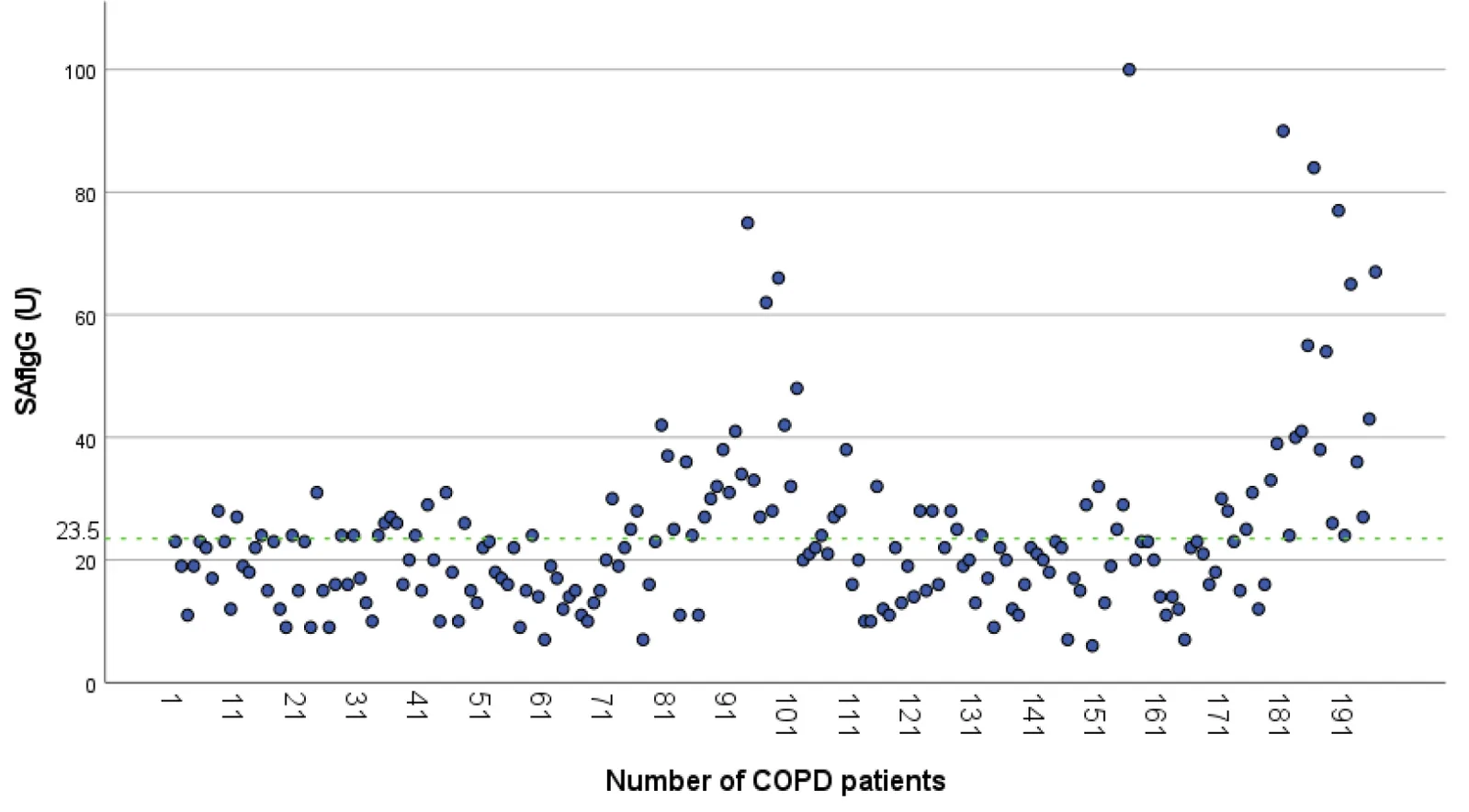
Distribution of serum *Aspergillus*-specific IgG levels in patients with chronic obstructive pulmonary disease

**Figure 2:**
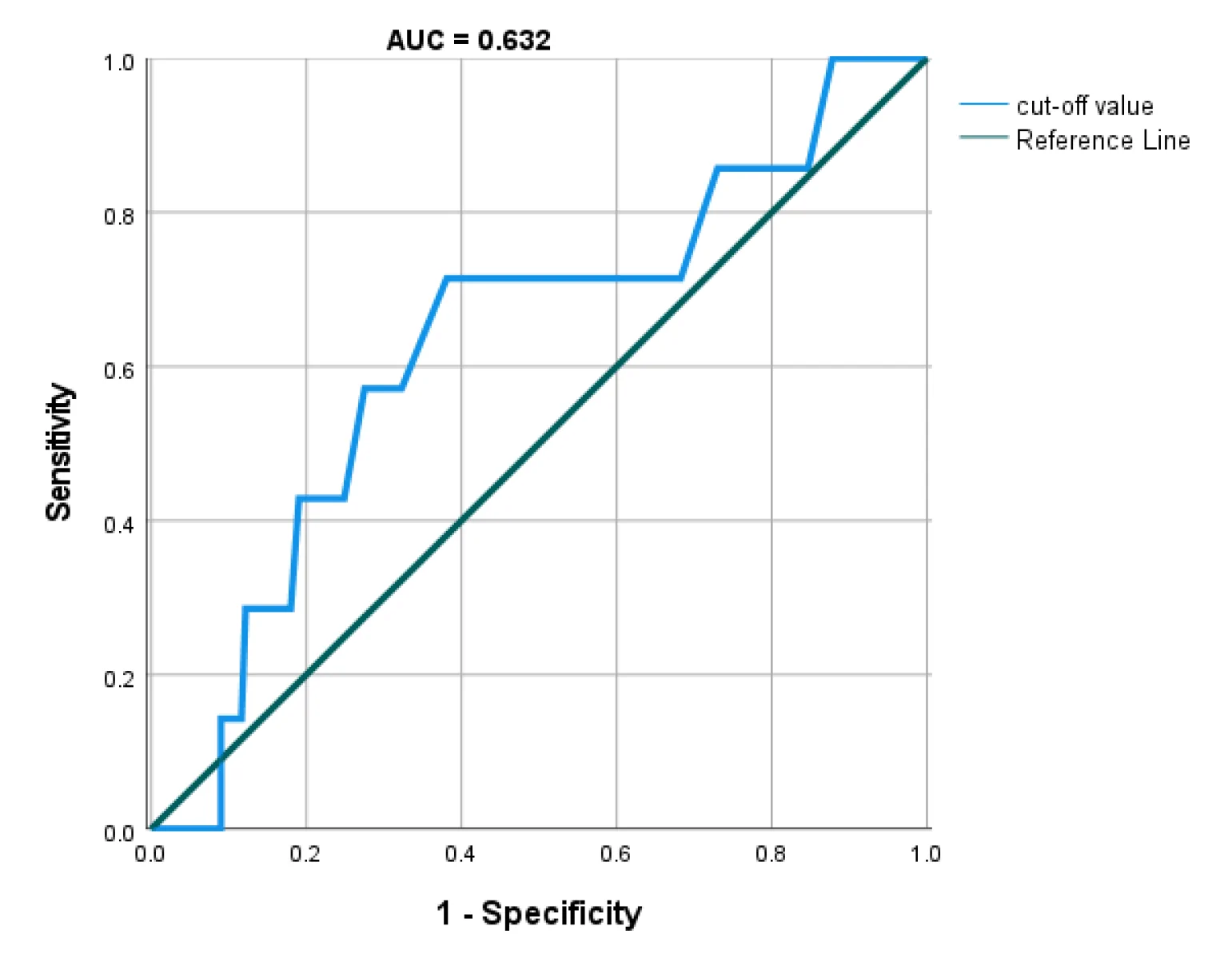
Receiver operating characteristic curve analysis of pulmonary disease

Cut-off value of 23.5 U yielded a sensitivity, specificity, PPV, NPV, and likelihood ratio of 71.4%, 61.4%, 6.4%, 98.3%, and 2.97, respectively. Based on the findings, all individuals who tested positive for *A. fumigatus* cultures were also positive for S*Af*IgG. In contrast, patients with positive cultures for *A. flavus* and *A. tubingensis* had S*Af*IgG levels of 22.1 U and 16 U, respectively. Based on the established S*Af*IgG cutoff levels in this study, 78 (39.8%) out of 196 COPD patients had positive S*Af*IgG results. The median S*Af*IgG levels were significantly higher in CPA patients at 39 U (31-55) compared to non-CPA patients at 20 U (15-24) (*p*<0.001) (Table 1).

**Table 1:** Demographics, clinical profile, and mycological findings of the study population

Demographic	Total (N=196)	Non-CPA (N=166)	CPA (N=30)	*p* value
Male gender, n (%)	154 (78.6%)	132 (79.5%)	22 (73.3%)	0.447
Median age (years), (IQR)	61 (48.2-67)	61 (47-67)	58.5 (50-69)	0.807
Culture, n (%)	7 (3.6%)	2 (1.2%)	5 (16.7%)	<0.001
*Aspergillus* species, n (%)				
*Aspergillus* fumigatus	3 (1.5%)	0	3 (10%)	
*Aspergillus* flavus	2 (1%)	1 (0.6%)	1 (3.3%)	
*Aspergillus* tubingensis	1 (0.5%)	1 (0.6%)	0	
*Aspergillus* citrinoterreus	1 (0.5%)	0	1 (3.3%)	
Median S*Af*IgG, (IQR)	22 (15-28)	20 (15-24)	39 (31-55)	<0.001
S*Af*IgG, n (%)				
≥ 23.5	78 (39.8%)	48 (28.9%)	30 (100%)	<0.001

CPA: chronic pulmonary aspergillosis. S*Af*IgG: *Aspergillus fumigatus*-specific IgG

Level of S*Af*IgG was significantly higher in COPD patients with different pulmonary conditions and radiological features compared to those without such conditions and features, particularly bronchiectasis (*p*<0.001), cavitation (*p*<0.001), aspergilloma (*p*=0.043), pericavitary fibrosis (*p*<0.001), consolidation (*p*<0.001), pericavitary infiltration (*p*=0.055), and patchy ground glass (*p*=0.030) (Figure 3).

**Figure 3:**
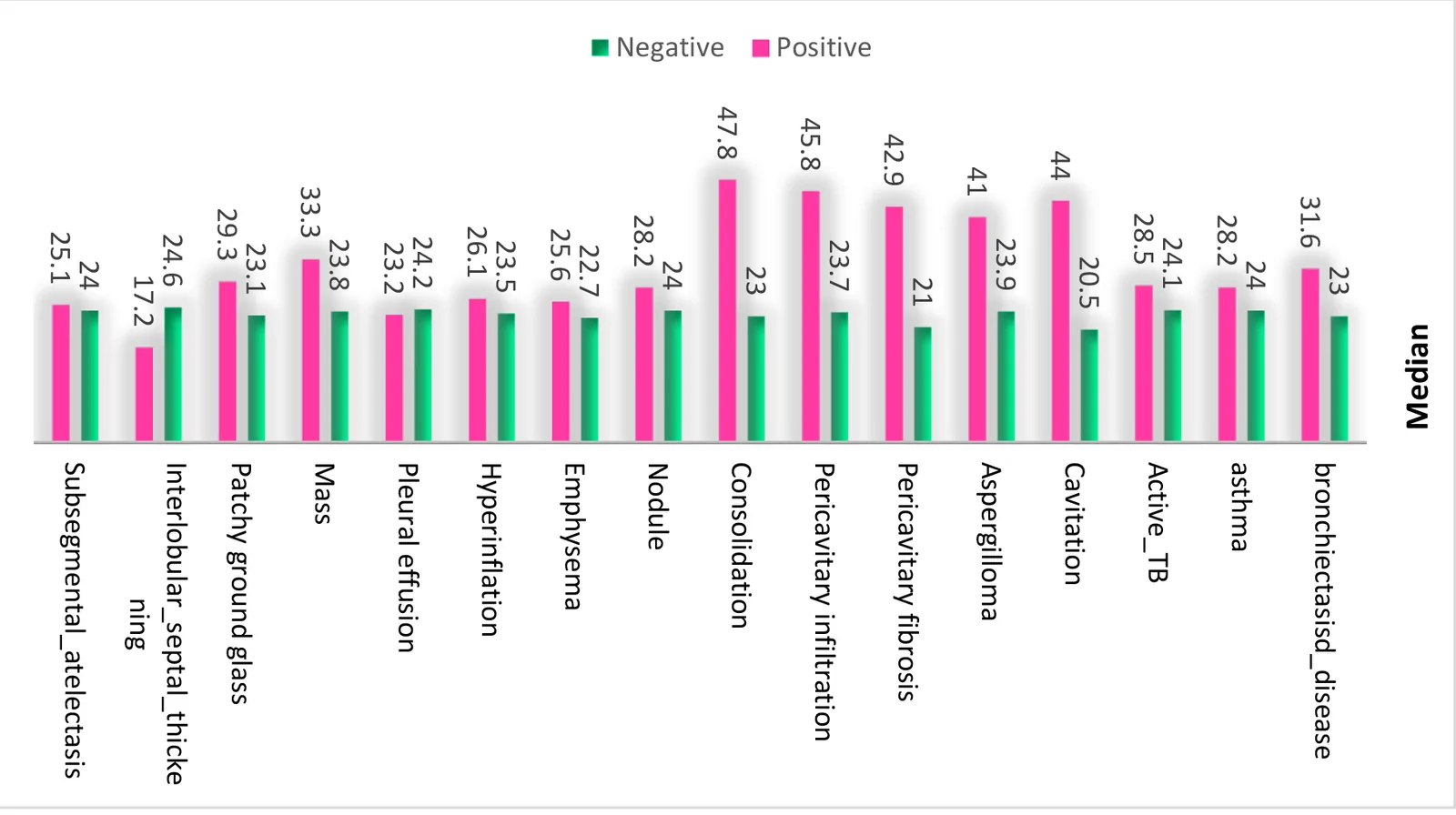
Distribution of the *Aspergillus fumigatus*-specific IgG level in chronic obstructive pulmonary disease patients with and without associated pulmonary condition and radiological features

## Discussion

This is the first report conducted in Iran to establish the optimal cut-off and evaluate the diagnostic performance of S*Af*IgG levels for CPA diagnosis. Due to the limited sensitivity of traditional mycological culture methods, there has been a shift towards S*Af*IgG assays for CPA diagnosis [7]. These assays are now recognized as pivotal diagnostic tools as they do not require invasive procedures for sample collection and offer improved sensitivity and specificity [2, 16]. However, it is important to note that the use of different diagnostic kits with varying cut-off values of S*Af*IgG [8, 9] presents a significant limitation, highlighting the need to determine appropriate cut-off levels for different geographical regions [7, 8].

The wide variation in reported cut-off values may influence the reported prevalence of CPA in different parts of the world [2, 9]. Accordingly, this study established a S*Af*IgG cut-off level of 23.5 U for the diagnosis of CPA in Iranian patients with COPD. In this study, S*Af*IgG had a sensitivity of 71.4%, but its specificity was relatively moderate at 61.4%. S*Af*IgG had a PPV of 6.4% and an NPV of 98.3%, indicating that the test can serve as an initial screening tool or be used in conjunction with other confirmatory tests, including the culture method. The results demonstrated that this test is primarily useful for ruling out disease due to its high NPV of 98.3%; however, it requires integration with clinical evidence or additional diagnostic tests to confirm a diagnosis, given its very low PPV of 6.4%.

Additionally, all patients who identified as having CPA by culture were also confirmed by the S*Af*IgG test. Notably, this cut-off is higher than the manufacturer's recommended diagnostic cutoff of 10 U, possibly linked to variations in the environmental distribution of *Aspergillus* spp. and the rate of individual exposure.

In a study using sera from 100 blood donors from Uganda and 241 CPA patients from the UK, Page et al. [8] evaluated the comparative performance of six assays with ROC curve analysis to determine the optimal diagnostic cutoff values. In line with the findings of the present study, they reported that cut-offs varied for each assay and differed from the recommendations of the manufacturers. In another study performed in Europe [17] using four different commercial assays, the same results were reported.

In the present study, 39.8% of COPD patients were positive for S*Af*IgG. It was also found that patients with CPA have higher levels of S*Af*IgG (median [IQR], 39 [31-55]) compared to non-CPA patients (median [IQR], 20 [15-24]). These results are consistent with those of another study performed by Palanivel et al. [18] in India and Hedayati et al. [19] in Iran, who reported a 29.5% and 44.3% sensitization rate to *Aspergillus* using S*Af*IgG detection in acute exacerbation of chronic obstructive pulmonary disease (AECOPD) and TB patients, respectively. In contrast to the study performed by Bafadhel et al. [20], which reported a 13% prevalence rate of S*Af*IgG in patients with AECOPD using the ImmunoCAP assay, the present study found a significantly higher rate of 70%.

The present study also revealed elevated S*Af*IgG levels among COPD patients with specific radiological presentations. In line with this research, a recent study highlighted the elevated S*Af*IgG levels in COPD patients with bronchiectasis (35.4 mg/L) compared to those without bronchiectasis (17.4 mg/L) [21]. Moreover, the present study reported a higher sensitivity for the S*Af*IgG assay, compared to only 16.7% for the culture method. Therefore, the diagnosis of CPA is made on the demonstration of S*Af*IgG along with radiological features due to the lower sensitivity of other microbiological methods to demonstrate *Aspergillus*. A difference in S*Af*IgG levels was observed between cases with positive cultures of *A. fumigatus* (median, 20) and those with positive cultures of non-*fumigatus* species (median, 30), although this difference did not reach statistical significance (*Pv*=0.157). Despite being close to the threshold of significance, this finding suggests that the S*Af*IgG detection kit may have specificity, which supports its diagnostic validity. However, these discrepancies highlight potential differences in patient populations or diagnostic methodologies that require further investigation. In cases where there were positive cultures for *A. flavus* and *A. tubingensis*, the S*Af*IgG level was below the diagnostic threshold and was recorded at 22.1 U and 16 U, respectively.

Non-*fumigatus Aspergillus* species may not express the specific antigens targeted by diagnostic kits designed exclusively for *A. fumigatus*, potentially leading to false-negative results. Although this emphasizes the high specificity and diagnostic reliability of S*Af*IgG detection kits, it also highlights an important limitation. Therefore, there is an urgent need to develop broader diagnostic assays that encompass antigens from a wider range of *Aspergillus* species to enhance diagnostic accuracy and improve patient management. In line with the findings of this research, a previous study has reported that infections caused by non-*fumigatus Aspergillus* species demonstrate lower cross-immunogenicity with *A. fumigatus*, resulting in decreased levels of S*Af*IgG [22].

A limitation of this study was the absence of a definitive CPA diagnosis based on histological evidence of *Aspergillus* infection in normally sterile tissue. Furthermore, only seven cases of culture-positive *Aspergillus* CPA were identified, which limited comparisons with diagnostic techniques that are not based on culture. A low number of culture-positive cases in our cohort (n=7, 3.6% of total) significantly reduced the statistical power of ROC analysis. This means that there may be a true association between S*Af*IgG and CPA that we were unable to detect due to the limitations of culture.

## Conclusion

The findings indicate that S*Af*IgG, with a cut-off value of 23.5 U, provides moderate sensitivity and specificity for screening of CPA diagnosis in COPD patients. It is also noteworthy that due to resource constraints and local practice, *Aspergillus* culture was the only readily available and standardized mycological test in the mycology laboratory of our country for CPA diagnosis. The authors are aware that culture has known limitations in sensitivity for the diagnosis of CPA. Therefore, it was assumed that the combination of two diagnostic approaches, namely culture and S*Af*IgG detection, can increase the CPA diagnosis and estimation of the true prevalence of CPA in different patients at risk. However, the variability in cut-off values across different populations highlights the need for standardized guidelines to improve diagnostic consistency and reliability.
